# Nontoxic singlet oxygen generator as a therapeutic candidate for treating tauopathies

**DOI:** 10.1038/srep10821

**Published:** 2015-06-01

**Authors:** Sahabudeen Sheik Mohideen, Yasutoyo Yamasaki, Yasuhiro Omata, Leo Tsuda, Yuji Yoshiike

**Affiliations:** 1 Alzheimer’s Disease Project Team, Center for Development of Advanced Medicine for Dementia, National Center for Geriatrics and Gerontology, 7-430 Morioka, Obu, Aichi, Japan 474-8511; 2 Department of Drug Discovery, Center for Development of Advanced Medicine for Dementia, National Center for Geriatrics and Gerontology, 7-430 Morioka, Obu, Aichi, Japan 474-8511; 3 Department of Occupational and Environmental Health, Graduate School of Medicine, Nagoya University, 65 Tsurumai-cho, Showa-ku, Nagoya, Japan 466-8550; 4 Animal Models of Aging, Center for Development of Advanced Medicine for Dementia, National Center for Geriatrics and Gerontology, 7-430 Morioka, Obu, Aichi, Japan 474-8511

## Abstract

Methylene blue (MB) inhibits the aggregation of tau, a main constituent of neurofibrillary tangles. However, MB’s mode of action *in vivo* is not fully understood. MB treatment reduced the amount of sarkosyl-insoluble tau in *Drosophila* that express human wild-type tau. MB concurrently ameliorated the climbing deficits of transgenic tau flies to a limited extent and diminished the climbing activity of wild-type flies. MB also decreased the survival rate of wild-type flies. Based on its photosensitive efficacies, we surmised that singlet oxygen generated through MB under light might contribute to both the beneficial and toxic effects of MB *in vivo*. We identified rose bengal (RB) that suppressed tau accumulation and ameliorated the behavioral deficits to a lesser extent than MB. Unlike MB, RB did not reduce the survival rate of flies. Our findings indicate that singlet oxygen generators with little toxicity may be suitable drug candidates for treating tauopathies.

Alzheimer’s disease (AD) is one of the most common forms of dementia. Cognitive dysfunction and memory loss are the main clinical symptoms of AD patients, while neurofibrillary tangles (NFTs) composed of aggregated tau protein are one of the pathological hallmarks. The extent of NFT formation correlates well with cognitive dysfunction in AD patients^1^. Therefore, one plausible therapeutic strategy for treating AD and other tauopathies would be to reduce the amount of NFTs in the brain.

Methylthioninium chloride, also known as Methylene Blue (MB), is a heterocyclic aromatic compound that inhibits tau aggregation *in vitro*^2^. MB treatment also reduces detergent-insoluble phosphorylated tau levels in P301L tau transgenic mice^3^. MB has also been found to reduce “soluble” tau^4^, and it rescues cognitive decline in rTg4510 transgenic mice^5^. In one study, reduction of total tau by MB was suggested to occur through proteasome-dependent tau degradation^4^. Another study indicates that MB-induced autophagic signaling reduces the level of total tau in tau-expressing CHO cells^6^.

*In vitro* MB oxidizes cysteine residues in Hsp72, resulting in reduced tau levels^7^. Recently, MB was reported to inhibit tau aggregation by oxidizing tau cysteine sulfhydryl groups to sulfenic, sulfinic, and sulfonic acids, converting both four-repeat and three-repeat tau to an aggregation-incompetent monomeric form^8^. Another group reported that MB inhibits four-repeat tau aggregation by oxidizing its two-cysteine residues through an alternative mechanism. Oxidation of two cysteine residues results in the formation of intramolecular disulfide bond that makes four-repeat tau fibrillization incompetent by preventing it from forming intermolecular disulfide bonds^9^. These studies provide *in vitro* evidence that the oxidizing properties of MB play an important role in inhibiting tau aggregation. Supporting this role, MB reportedly prevents tau-related neurotoxicity in P301S transgenic mice by upregulating the antioxidant response pathway through NF-E2-related factor 2 (Nrf2/ARE)^10^.

Previously, we investigated the effects of alloxan, a glucose analog and oxidizing compound, on tau accumulation in transgenic mice^11^. Low-dose alloxan treatment reduces the accumulation of both soluble and insoluble tau in these transgenic mice, without inducing apparent diabetic symptoms. However, alloxan treatment using a higher, diabetogenic dose promotes the phosphorylation of tau. Together with the low-dose results, these results suggest there is a dose-dependent effect of oxidation on the fate of tau *in vivo*.

MB also is known to have dose-dependent effects *in vivo* and *in vitro*. When treated with moderate MB doses, rats show a high level of wheel-running activity, whereas at low or high doses rats show a lower level wheel-running activity compared to control rats^12^. Low-dose MB treatment destabilizes tau aggregation in SH-SY5Y cells, while a high-dose of MB induces severe cellular damage^13^. High-dose MB treatment induces hemolysis due to hemoglobin oxidation^14^. Although high doses of MB also induce methemoglobinemia, low doses are used to treat methemoglobinemia^15^,^16^. In a phase-II clinical MB trial for possible treatment of AD, three doses of MB were used. Treatment with two lower doses (30 mg or 60 mg MB capsules, three times a day) for 24 weeks effectively inhibited disease progression, as suggested by suppressing further cognitive dysfunction^17^,^18^. The highest dose (100 mg MB capsules, three times a day), however, was ineffective^18^. The lack of an effect at a high-dose MB was assumed to be indirect, partially due to limitation in the ability to absorb MB, depending on its redox state, in stomach in the presence of food^18^.

Taken together, these results suggest the role of oxidizing property of MB with regard to its perplexing impacts. To gain more insights on how MB works *in vivo*, we decided to use a *Drosophila melanogaster* model, one in which human tau protein is expressed. The major advantage of using this model is that flies have a short lifespan, allowing the effects of transgenic tau expression to be assessed in a reasonably short time.

The fruit fly, *Drosophila melanogaster,* has been successfully used to better understand certain aspects of animal and human behavior, circadian rhythms, and learning and memory, to name a few. In particular, genetic manipulation of protein expression through the GAL4-UAS system in this model has revealed key insights into human disease. This system allows for the expression of a target gene downstream of an upstream activator sequence (UAS) driven by the tissue-specific expression of yeast GAL4 transcription factor^19^. Transgenic proteins expressed in all fly neurons can induce neurotoxicity; thus, those flies can be used as a model to assess the effects of drugs on protein accumulation and their phenotypes. Another advantage of using *Drosophila* is that we are able to evaluate the toxicity of drug treatment *in vivo* by analyzing mortality and behaviors.

Expressing human mutant tau in *Drosophila* neurons shortens lifespan, induces brain vacuolization, causes loss of olfactory learning, and causes climbing deficits^20^,^21^,^22^. Even though NFTs cannot be histopathologically demonstrated in the human-tau fly model^20^, we confirmed the presence of accumulated tau biochemically by detecting tau in the sarkosyl-insoluble fraction of samples derived from fly heads. By treating this fly model with MB, we were able to examine the effects of MB on tau accumulation and associated phenotypes. MB treatment effectively reduced both soluble and insoluble-tau accumulation, but it caused toxic effects as well. In search of a compound that has a common efficacy as MB on tau, but with a better safety profile, we investigated the effects of rose bengal (RB). RB functions as an oxidant by generating a specific form of a non-free radical reactive oxygen species (ROS) called singlet oxygen, a property shared with MB^23^,^24^.

## Results

### MB treatment effectively reduces tau in sarkosyl-insoluble and TBS-soluble fractions of GMR;hTau flies

To date, no AD-like pathological hallmarks, such as NFTs, have been observed microscopically in flies expressing human tau^20^. Aggregated tau proteins become insoluble in the detergent sarkosyl^25^, and thus can be reasonably used as a proxy measure of “pathology.” In the present study, we successfully detected tau accumulation in sarkosyl-insoluble fractions extracted from the heads of flies that express human wild-type tau.

**Figure 1:**
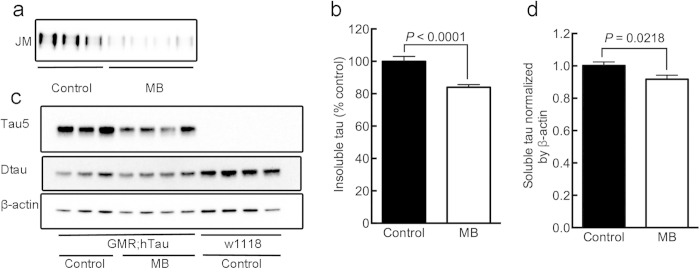
MB treatment reduces tau levels in sarkosyl-insoluble and TBS-soluble fractions from GMR;hTau flies. After one-month MB (1 mM) treatment, TBS-soluble and sarkosyl-insoluble fractions were prepared from the heads and analyzed by Western blot.(**a**) Western blot showing pan-tau JM antibody immunoreactivity of the sarkosyl-insoluble fraction. (**b**) Quantification of JM immunoreactivity of sarkosyl-insoluble tau showing that MB reduced tau levels (*P* < 0.0001). (**c**) Western blots showing immunoreactivity of the TBS-soluble fraction extracted from GMR;hTau and untreated wild-type *W*^*1118*^ flies. (**d**) Quantification of tau5 immunoreactivity of the TBS-soluble fraction showing that MB reduced soluble human tau levels (*P* = 0.0218). Quantitative data are means ± SEM of control (n = 17) and MB-treated groups (n = 15) of GMR;hTau and untreated, control wild-type *W*^*1118*^ flies (n = 4). Blots were cropped as shown in the figure within the rectangular box to represent the bands that were reactive to the pan-human tau antibodies (JM and tau5) at 65 kDa, *Drosophila* tau antibody (Dtau) at 50 kDa, and β-actin antibody at 42 kDa, respectively. Note that tau5 shows immunoreaction only in GMR;hTau fly samples but Dtau shows immunoreaction in both GMR;hTau and wild-type fly samples. Soluble tau levels were normalized by the band intensity of β-actin used as a loading control. All the gels were run under the same experimental conditions. The full-length blots of the cropped blots for Fig. 1 are available in the Supplementary Figure S1.

We first determined whether MB treatment affects tau accumulation in a GMR;hTau fly model, which expresses tau in the eye. MB applied in this *in vivo* fly model significantly reduced the amount of human tau (hTau) in the sarkosyl-insoluble fraction (*P* < 0.0001) (Fig. 1a,b), suggesting that MB suppresses tau aggregation also in this *in vivo* model. A reduction in tau level was also observed in the TBS-soluble fraction (*P* = 0.0218) (Fig. 1c,d). Such a reduction was not clearly detected in the level of endogenous *Drosophila* tau (Fig. 1c). We next examined how MB treatment influences functional behaviors, such as the climbing deficits reported for ELAV;hTau flies. These flies express hTau in all their neurons^21^.

### MB treatment suppresses tau accumulation and concurrently ameliorates climbing deficits in human tau expressing flies

Wild-type *Drosophila* instinctively climb upward against gravity, a phenomenon known as negative geotaxis^26^. We quantified climbing ability of wild-type flies, ELAV;hTau flies, and wild-type and ELAV;hTau flies treated with MB (see **Methods**).

**Figure 2:**
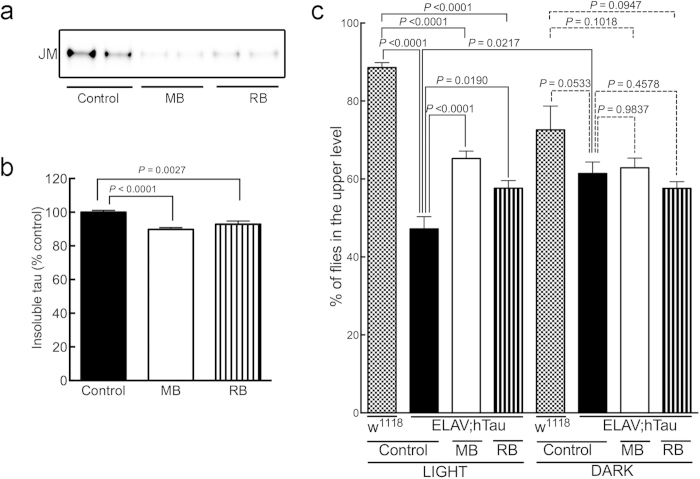
MB treatment suppresses tau accumulation and concurrently ameliorates climbing deficits of ELAV;hTau flies. Prior to head dissection, climbing activity was evaluated, as described in **Methods.** (**a**) Western blots showing JM immunoreactivity of sarkosyl-insoluble fractions derived from the heads of control (untreated), MB-treated, and RB-treated ELAV;hTau flies. (**b**) Quantification of JM immunoreactivity of sarkosyl-insoluble tau showing that both MB and RB treatment reduced insoluble tau levels (*P* < 0.0001 and *P* = 0.0027, respectively). (**c**) Percentage of ELAV;hTau and wild-type *w*^*1118*^ flies that climbed higher than 3 cm (upper level) from the bottom of the vial. This shows that MB and RB ameliorated the climbing deficits of ELAV;hTau flies (*P* < 0.0001 and *P* = 0.0190, respectively). Note that MB is more effective than RB in both reducing insoluble tau levels and ameliorating climbing deficits. The climbing activity of untreated control ELAV;hTau fly improved significantly (*P* = 0.0217, control in light *vs.* control in dark) when kept in darkness, “dark” condition, for one month, resulting in the statistical insignificance of MB-treatment effect (*P* = 0.9837, control in dark *vs.* MB in dark) and of RB-treatment effect (*P* = 0.4578, control in dark *vs.* RB in dark). In (b), data are means ± SEM of control (n = 17), MB-treated (n = 13), and RB-treated (n = 15) groups. In the climbing assay, data are means ±SEM of untreated, control (n = 39), MB-treated (n = 39), and RB-treated (n = 39) groups for the “light” conditions; and untreated, control (n = 10), MB-treated (n = 8), and RB-treated (n = 7) groups for the “dark” conditions. For comparisons, untreated wild-type flies in “light” (n = 7) and in “dark” (n = 5) were included. The blot was cropped as shown in the figure within the rectangular box to represent the bands that were reactive to the pan-human tau antibodies at 65 kDa. All the gels were run under the same experimental conditions. The full-length blots of the cropped blots are available in the Supplementary Figure S2.

ELAV;hTau flies at one month old displayed severe climbing deficits compared to wild-type flies (*P* < 0.0001) under normal light cycle (Fig. 2c). One month of 1 mM MB treatment not only significantly reduced the amount of tau in the sarkosyl-insoluble fraction derived from ELAV;hTau fly heads (*P* < 0.0001) (Fig. 2a,b), but also ameliorated the climbing deficits (*P* < 0.0001) (Fig. 2c). However, the degree of amelioration in the climbing activity of ELAV;hTau flies by MB treatment was quite limited as seen in the substantial gap between MB-treated ELAV;hTau and untreated wild-type flies (*P* < 0.0001) (Fig. 2c). This phenomenon is further discussed later in more detail.

**Figure 3:**
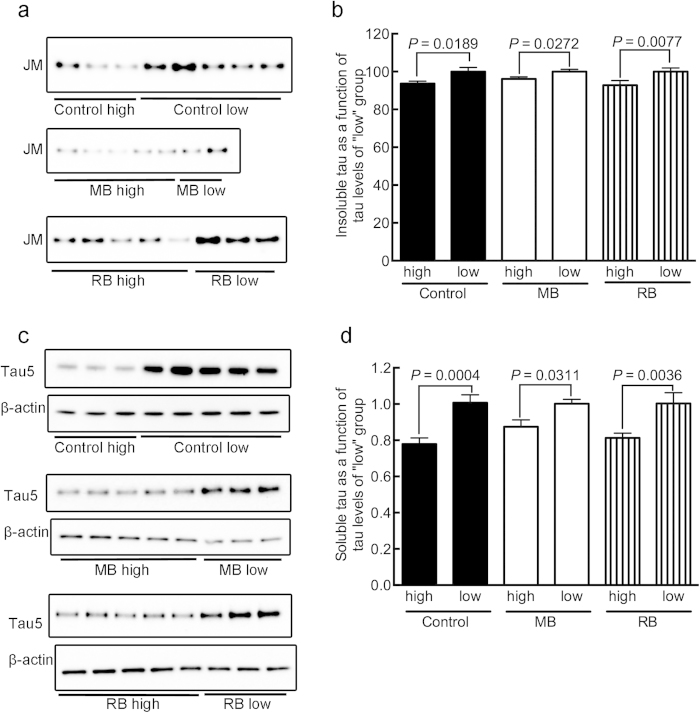
Climbing deficits in ELAV;hTau flies were correlated with the accumulation of insoluble and soluble tau. Flies that climbed higher than 3 cm from the bottom of the vial (high) and flies that could not climb (low) were separated and counted before their heads were dissected for biochemical analysis. (**a**) Western blot showing JM immunoreactivity of sarkosyl-insoluble fractions from “high” and “low” climbing flies. (**b**) Quantification of JM immunoreactivity of sarkosyl-insoluble tau showing that control, MB-treated, and RB-treated flies that climbed higher than 3 cm had significantly less tau than the flies that could not climb above the 3 cm level in each group (control, *P* = 0.0189; MB-treated, *P* = 0.0272; and RB-treated, *P* = 0.0077). (**c**) Western blot showing tau5 and β-actin immunoreactivities of TBS-soluble fractions from “high” and “low” climbing flies. (**d**) Quantification of tau5 immunoreactivity of TBS-soluble tau showing that control, MB-treated, and RB-treated flies that climbed higher than 3 cm had significantly less tau than the flies that could not climb above the 3 cm level in each group (control, *P* = 0.0004; MB-treated, *P* = 0.0311; and RB-treated, *P* = 0.0036). In the biochemical analysis, data are means ±SEM of control (n = 16 for both “high” and “low” flies); MB-treated (n = 18 and n = 10 for “high” and “low” flies, respectively); and RB-treated (n = 21 and n = 10 for “high” and “low” flies, respectively). Note that the average of the “low” group was set to 100% for each treatment condition. Blots were cropped as shown in the figure within the rectangular box to represent the bands that were reactive to the pan-human tau antibodies at 65 kDa. Soluble tau level was normalized by the band intensity of β-actin used as a loading control. All the gels were run under the same experimental conditions. The full-length blots of the cropped blots are available in the Supplementary Figure S3.

To better understand the relationship between tau accumulation and the severity of the climbing deficit in ELAV;hTau flies, we measured the amount of insoluble tau in flies that could climb higher than 3 cm and those that could not. ELAV;hTau flies that climbed higher than 3 cm (“high” in Fig. 3) had less head-derived sarkosyl-insoluble tau than those that could not reach this height (“low” in Fig. 3) (*P* = 0.0189, control high *vs.* control low) (Fig. 3a,b). This trend also applied to flies treated with compounds. However, the ratio of the number of MB-treated flies that could climb higher than the 3 cm to those that could not reach this height was greater than that of the untreated control group (Fig. 3a). These results suggest that the climbing deficits in flies that overexpress tau in neurons are directly correlated with, and could be caused by, tau accumulation and the amount of tau accumulated. It is tempting to speculate that this phenomenon observed in our fly model parallels the observation observed in AD patients, that the degree of cognitive decline prior to death is positively correlated with the extent of NFT formation^1^. What makes the fly model further interesting is that there was a significant difference in the soluble tau levels between “high” and “low” as well (*P* = 0.0004, control high *vs.* control low) (Fig. 3c,d), supporting a potential pathogenic role of soluble tau as suggested previously^27^.

### MB treatment can also lead to detrimental effects in flies

**Figure 4:**
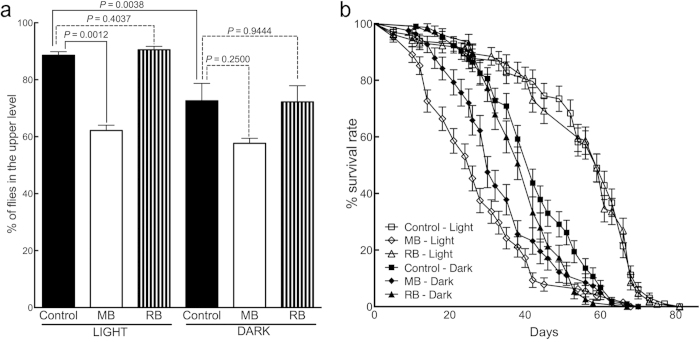
MB treatment also induces detrimental effects. Wild-type w1118 flies were treated with MB or RB (see Compound Treatment, Climbing Assay, and Survival Assay in Methods). (**a**) Quantification of climbing activity of *w*^*1118*^ flies after MB or RB treatment. Percentage of flies that climbed 3 cm above the bottom of the vial was significantly reduced (*P* = 0.0012) after MB but not RB treatment (*P* = 0.4037) under normal light cycle, “light” condition. Control *w*^*1118*^ flies received no compound treatment, only agar. The climbing activity of control fly declined significantly (*P* = 0.0038, control in light *vs.* control in dark) when kept in darkness, “dark” condition, for one month, resulting in the statistical insignificance of MB-treatment effect (*P* = 0.2500, control in dark *vs.* MB in dark). (**b**) Survival curves of *w*^*1118*^ flies showing the survival rate of wild type *w*^*1118*^flies treated with MB or RB. Control *w*^*1118*^ flies received no compound treatment. Under normal light cycle, MB treatment significantly decreased the survival rate of wild-type flies (*P* < 0.0001, control in light *vs.* MB in light). Such a toxic effect was significantly reduced in continuous “dark” condition (*P* < 0.0233, MB in light *vs.* MB in dark). The survival rate after RB treatment was not significantly different from control flies in “light” condition (*P* = 0.8816). For the climbing assay (**a**), data are means ± SEM of control (n = 7), MB-treated (n = 6), and RB-treated (n = 8) groups under “light” condition and control (n = 5), MB-treated (n = 3), and RB-treated (n = 5) groups under “dark” condition. Survival assay (**b**), the final counts for each “light” group were: control, n = 98 flies; MB-treated, n = 128 flies; RB-treated, n = 104 flies and those for each “dark” group were: control, n = 103 flies; MB-treated, n = 82 flies; RB-treated, n = 75 flies. Each data point is the mean number of surviving flies (±SEM) on each day.

Apparently, MB treatment suppressed tau accumulation, leading to the amelioration of climbing deficits in tau-expressing flies. However, the efficacy of MB in improving the flies’ climbing ability was limited, since more than a few MB-treated flies failed to climb higher than 3 cm (Fig. 2c). We next treated wild-type *w*^*1118*^ flies with 1 mM MB for one month and examined their climbing ability. MB treatment significantly reduced the proportion of *w*^*1118*^flies that climbed higher than the 3 cm mark (*P* = 0.0012) (Fig. 4a). The effects of MB-treatment on wild-type flies suggests one possibility for why the extent of improvement in the climbing activity of ELAV;hTau by MB treatment was limited (P < 0.0001, untreated *w*^*1118*^ vs. MB-treated ELAV;hTau) (Fig. 2c). As a matter of the fact, the level of climbing activity in ELAV;hTau flies being treated with MB (65.3%) (Fig. 2c) is close to the level in MB-treated *w*^*1118*^ flies (62.2%) (Fig. 4a) (*P* = 0.2812). Thus, it is conceivable that the toxic impact of MB treatment held down its improvement effect on the climbing activity of fly model.

During the experiments, we noticed that some flies in the MB-treatment group died rather quickly. To examine this further, we measured the survival rate of MB-treated *w*^*1118*^ flies. Indeed, our MB treatment protocol significantly reduced the survival rate of *w*^*1118*^ flies (*P* < 0.0001, control in light *vs.* MB in light) (Fig. 4b). These results clearly indicate that one month of 1 mM MB treatment not only has beneficial effects, as shown in the other experiments with hTau flies, but also has detrimental effects. Nonetheless, the climbing ability of ELAV;hTau flies and wild-type flies could be used as an index for evaluating the effect of a compound on both tau accumulation (i.e., efficacy) and natural fly behavior by negative geotaxis (i.e., toxicity), respectively so that these flies would make a good screening system for compounds safely reducing tau accumulation.

### MB treatment may ameliorate tau accumulation by promoting responses to the singlet oxygen generated via photoactivation

How could MB cause both beneficial and detrimental effects *in vivo*? MB is known for its hormetic, non-linear, dose-dependent effects^16^. For example, high doses of MB induce methemoglobinemia, while low doses are used to treat it. This hormetic dose-response effect is presumably due to the redox activity of MB^15^,^16^. Specifically by means of its oxidizing properties, MB is thought to damage hemoglobin, DNA, and ribosomes^14^,^28^,^29^ On the other hand, MB-associated oxidation has been shown to inhibit tau protein aggregation *in vitro*^8^,^9^. Our previous study with alloxan also suggests that low levels of oxidation and mild oxidative stress suppress tau accumulation in the brains of murine models^11^. Therefore, we deduced that oxidation induced by MB might also suppress tau-associated phenotypes in fly models.

**Figure 5:**
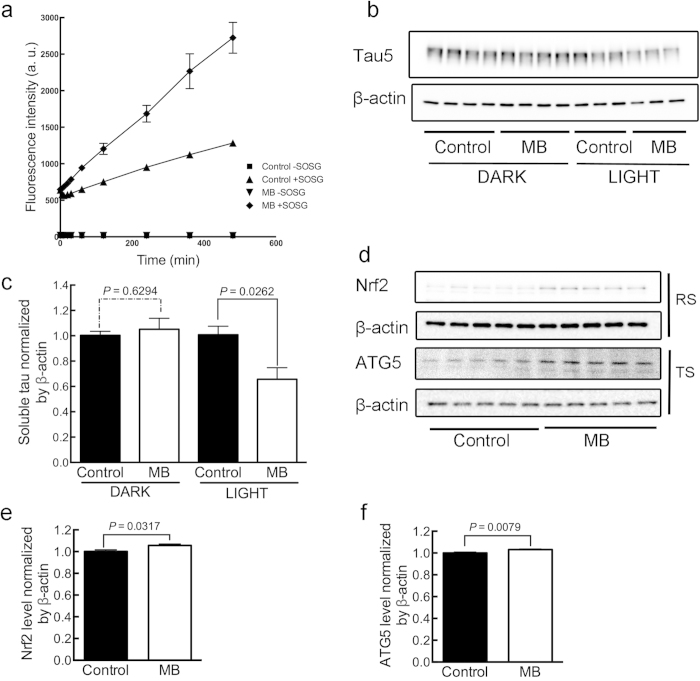
Under light MB generates singlet oxygen that promotes oxidative stress responses and autophagy. (**a**) Measurement of singlet oxygen generating from homogenates of GMR;hTau flies that were treated with 1 mM MB agar or control agar for two weeks using SOSG. After 8h of incubation, the generation of singlet oxygen was significantly greater in homogenates of flies fed with MB than the untreated control (control + SOSG *vs.* MB + SOSG, *P* = 0.0047). (**b**) Western blots showing pan-tau tau5 and β-actin immunoreactivities of TBS-soluble fraction of GMR;hTau flies that were treated for two weeks with 1 mM MB agar or control agar under either continuous darkness or normal light cycle conditions (12h:12h). (**c**) Quantification of tau5 immunoreactivity of TBS-soluble tau showing that MB treatment significantly reduced TBS-soluble tau levels only in the presence of light. (**d**) Western blots showing Nrf2 and β-actin immunoreactivities of RIPA-soluble (RS) fractions and ATG5 and β-actin immunoreactivities of TBS-soluble (TS) fractions of GMR;hTau flies that were treated with 1 mM MB agar or control agar under normal light cycle for two weeks. Quantification of (**e**) Nrf2 and (**f**) ATG5 immunoreactivities of RS and TS fraction respectively, showing that MB treatment significantly increased Nrf2 and ATG5. Statistical analyses were conducted using two-way repeated measures ANOVA in (a) and the Mann-Whitney test in (c), (e) and (f). In (a), data are means ±SEM of control –SOSG (n = 3), control + SOSG (n = 3), MB-treated –SOSG (n = 3), MB-treated + SOSG (n = 3). In (c), data are means ±SEM of control (n = 8) and MB-treated (n = 8) groups under “dark” conditions and control (n = 7) and MB-treated (n = 7) groups under “light” conditions. In (e) and (f), data are means ±SEM of control (n = 5) and MB-treated (n = 5) groups under “light” conditions. Soluble human-tau, Nrf2, and ATG5 levels were normalized by β-actin used as a loading control in the respective fraction. All the gels were run under the same experimental conditions. The full-length blots of the cropped blots are available in the Supplementary Figure S4.

MB is a photosensitizer^30^. Its oxidizing effects are at least in part due to a specific form of non-free radical ROS called singlet oxygen, which is generated when MB is exposed to light^23^,^24^. We next measured the amount of singlet oxygen in untreated or MB-treated fly homogenates under light by using SOSG (Singlet Oxygen Sensor Green reagent) (see **Compound treatment** and **Singlet Oxygen measurement** in **Methods**) (Fig. 5a). In the figure, “−SOSG” indicates measurement in the absence of SOSG reagent and “+SOSG” indicates measurement in the presence of SOSG reagent. Fluorescence counts show the generation of singlet oxygen. The increase in the amount of singlet oxygen over time was substantially greater in homogenates from flies treated with MB (control + SOSG *vs.* MB + SOSG, *P* = 0.0047), confirming the absorption of MB by flies fed with MB-containing agar. Although this result does not directly show the generation of singlet oxygen *in vivo*, at least it suggests a potential of generating singlet oxygen in the brains of MB-treated flies. If singlet oxygen generated via photoactivation plays a role in the suppression of tau accumulation, then the tau-reducing effect of MB should be affected by the presence or absence of ambient light. MB treatment (1 mM) of GMR;hTau flies for two weeks reduced tau when maintaining the flies under a normal light cycle (*P* = 0.0262), but MB did not when maintaining the flies under continuous darkness (*P* = 0.6294) (Fig. 5b,c). The part of light was also examined on the climbing activity and the survival rate of flies (Figs. 2 and 4) The absence of light itself affected severely the climbing activity of flies. ELAV;hTau flies showed better mobility when kept in “dark” than “light” (*P* = 0.0217, control in light *vs.* control in dark) (Fig. 2c). This could be due to the reduced accumulation of insoluble hTau in continuous dark over one month for reasons yet to be revealed (Supplementary Figure S2). As a result, the beneficial effect of MB treatment was not observed in the “dark” condition (*P* = 0.9837, control in dark *vs.* MB in dark) (Fig. 2c). Keeping wild-type flies in darkness for 1 month, on the other hand, suppressed their climbing activities (*P* = 0.0038, control in light *vs.* control in dark) (Fig. 4a). This could be induced by the disrupted circadian rhythms as reported^31^. Note that the combination of improved climbing activity in ELAV;hTau flies and suppressed climbing activity in wild-type flies made the difference in their climbing activities statistically insignificant (*P* = 0.0533, control *w*^*1118*^ in dark *vs.* control ELAV;hTau in dark) under continuous dark condition (Fig. 2c). Lowered mobility in wild-type flies by “dark” treatment also made the detrimental effect of MB treatment undetectable (*P* = 0.2500, control in dark *vs.* MB in dark) (Fig. 4a). Continuous darkness lowered not only the climbing activity of wild-type flies but also their survival rates (*P* < 0.0001, control in light *vs.* control in dark) (Fig. 4b). With regards to this subject, there is a great deal of evidence showing the relationships between circadian systems and longevity particularly in flies^32^,^33^. In the mean time, the suppression of survival rate by MB treatment was ameliorated if flies were kept in dark continuously (*P* = 0.0233, MB in light *vs.* MB in dark) although there remained certain degree of toxicity (*P* = 0.0007, control in dark *vs.* MB in dark), suggesting detrimental effects of MB treatment independent of light (Fig. 4b). In sum, these results suggest that photosensitivity affects largely on tau accumulation, climbing activity, and the survival rate of flies. But, what are the molecular bases? We found some clues in that a protein Nrf2 (CncC, *Drosophila* orthologue) known for its roles in oxidative stress responses was increased by two-week MB treatment on GMR;hTau flies (*P* = 0.0317), suggesting an induction of anti-oxidant defense mechanisms (Fig. 5d,e). One such mechanism might be autophagy as its constitutive protein ATG5 was increased as well (*P* = 0.0079) (Fig. 5d,f). Inductions of both oxidative stress responses and autophagy by MB have also been reported previously in other systems^6^,^10^.

### RB treatment modestly reduces tau accumulation and safely ameliorates behavioral deficits in tau fly models

Because MB also has detrimental effects, we searched for a compound that shares the singlet oxygen-generation property of MB but has a better safety profile. One photosensitizer compound that met these criteria was a food additive called rose bengal or RB^34^,^35^. We treated ELAV;hTau flies for one month in agar containing 1 mM RB and found that RB treatment reduced the accumulation of sarkosyl-insoluble tau (*P* = 0.0027) (Fig. 2a,b).

The climbing activity of these ELAV;hTau flies was also improved (*P* = 0.0190, control in light *vs.* RB in light) (Fig. 2c). In terms of efficacy, RB was less potent than MB (Fig. 2c). Similar to the case of MB treatment, this beneficial effect of RB treatment was masked in the “dark” condition (*P* = 0.4578, control in dark *vs.* RB in dark) (Fig. 2c). Unlike MB, RB treatment did not affect the climbing activity of wild-type *w*^*1118*^flies (*P* = 0.4037 in light and *P* = 0.9444 in dark, control *vs.* RB) (Fig. 4a). Moreover, the effect of RB treatment on the survival rate of wild-type *w*^*1118*^flies was insignificant (*P* = 0.8816) and RB-treated flies held higher survival rate than MB-treated flies (*P* < 0.0001) under normal light cycle (Fig. 4b). These results suggest that RB is less toxic to flies than MB and that the lower potency of RB to improve climbing activity than MB is unlikely due to the toxicity of RB treatment.

In the fly model, climbing activity was directly correlated with the extent of tau accumulation that was reduced by MB or RB. How compound treatment affects on cognitive function in flies remains unknown. To test the effect of RB treatment on memory, we used mb;hTau flies that overexpress tau specifically in the mushroom body (mb), a brain area of the fly known to be associated with olfactory learning and memory^36^,^37^.

**Figure 6:**
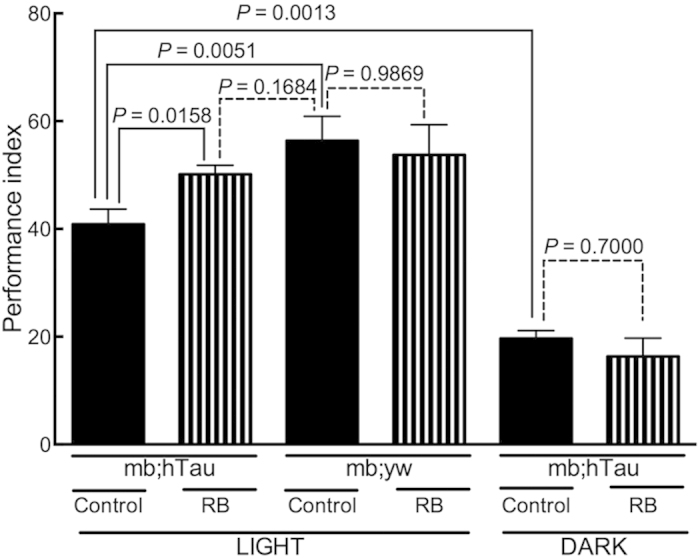
RB ameliorates olfactory memory deficits in mb;hTau flies. Olfactory memory assay (larger numbers are better performance; see **Olfactory memory assay** in **Methods**). Performance index (PI) of the mb;hTau flies was significantly less than that of mb;yw flies (*P* = 0.0051). One-week of RB treatment significantly improved the olfactory memory of mb;hTau flies (*P* = 0.0158, control in light *vs.* RB in light). Such an improvement was not detected when flies were kept in continuous darkness during treatment (*P* = 0.7000, control in dark *vs.* RB in dark), as the performance of untreated mb;hTau flies was worsened in contrast to the flies kept under normal light cycle (*P* = 0.0013, control in light *vs.* control in dark). Statistical analyses were conducted using the Mann-Whitney test. Data are means ±SEM of control (n = 22) and RB-treated (n = 22) groups of mb;hTau flies and control (n = 11) and RB (n = 11) groups of mb;yw flies under “light” conditions and control (n = 3) and RB-treated (n = 3) groups of mb;hTau flies under “dark” conditions.

Expression of hTau in the mb induces deficits in olfactory learning and memory in a fly model of tauopathy^22^. Thus, we examined the effect of RB treatment on associative memory between a specific odor and electrical shocks according to the procedures described in the **Methods** section. Treating mb;hTau flies with 1 mM RB for one week improved short-term olfactory memory (*P* = 0.0158, control in light *vs.* RB in light) (Fig. 6). In the condition where the olfactory memory was further suppressed under continuous darkness (*P* = 0.0013, control in light *vs.* control in dark), the improvement effect of RB treatment could not be seen (*P* = 0.7000, control in dark *vs.* RB in dark). We were unable to evaluate the effect of MB treatment on olfactory memory, because MB greatly increased mortality among mb;hTau flies.

Although we did not thoroughly examine the dose-response effects of MB and RB, it is conceivable that treating flies with different doses of these compounds would nonlinearly change the extent of their beneficial and detrimental effects. In terms of benefit, treatment with 1 mM RB also reduced tau accumulation and ameliorated behavioral deficits like MB, but to a smaller extent.

Singlet oxygen is responsible for the photosensitive oxidizing property commonly possessed by MB and RB, and thus it might play a crucial role in reducing tau accumulation. Therefore, we propose that compounds generating singlet oxygen with little adverse effects *in vivo* may be promising therapeutic candidates for the treatment of tauopathies, including AD.

## Discussion

The number of AD patients is increasing dramatically worldwide, as there is no effective way to cure this devastating disorder. A way to prevent or stop the progression of AD is urgently needed. Several drugs targeting amyloid β have been tried previously, and some still have great potential. Lately, candidate drugs targeting tau have gained much attention and expectations remain high. MB is the first tau-targeted drug that was demonstrated to inhibit tau aggregation *in vitro*^2^. In a phase II clinical trial, MB at low and moderate doses effectively slowed the progression of cognitive decline in AD patients^17^,^18^. At the highest dose, however, MB was ineffective after treatment for 24 weeks^18^. The use of MB has proceeded to the phase III stage of assessment, even though its mode of action on tau *in vivo* is incompletely understood. One of our aims in this study was to fill in some of these gaps for obtaining alternative drug seed against tauopathies.

In *Drosophila* models of tauopathy, we were able to detect tau biochemically in sarkosyl-insoluble fractions derived from the heads of flies expressing wild-type human tau (Figs. 1a and 2a). Thus, in the present study we used *Drosophila* models of tauopathy to investigate the effects of MB on tau accumulation and certain behavioral phenotypes *in vivo*. MB treatment reduced not only the accumulation of tau in fly heads but also ameliorated their behavioral deficits (Fig. 2). However, it failed to completely ameliorate the climbing deficits of tau-expressing flies (Fig. 2c) and, in wild-type flies, climbing ability worsened and the survival rate became lower after MB treatment (Fig. 4).

The mechanism by which MB induces both beneficial and detrimental effects is still being determined. However, one possibility may involve autophagy and the oxidizing ability of MB for two reasons: (1) MB treatment has been shown to mitigate tauopathy by inducing autophagy^6^; and (2) autophagic pathways, which are induced by oxidation^38^,^39^, degrade oxidized proteins^40^. In the MB-treated flies, we also observed indications of oxidative stress response such as Nrf2 upregulation and autophagy such as ATG5 (Fig. 5d–f). Therefore, it is conceivable that MB potently induces autophagy due to its oxidation property, thus enhancing rather nonspecific degradation of organelles and molecules including hTau that is excessively abundant in fly models but not in wild type.

Under *in vivo* conditions, MB has a hormetic dose-dependent effect due to its redox properties^15^,^16^. MB at high doses causes methemoglobinemia, while MB at low doses is used to treat methemoglobinemia^16^. The inefficacy of treatment at the highest dose during the phase II trial was suggested to be due in part to the oxidized MB in both cross-linking of the gelatin capsule shells combined with the increasing insolubility of the drug suspension and the extra conversion step to the reduced form to be absorbed in stomach that is interfered by the presence of food^18^. It was also suggested that oxidized MB absorbed from the small intestine have a greater propensity to oxidize hemoglobin^18^. In terms of delivering issues and side effects, the reduced form of MB appears to be a better choice than the oxidized form. Paradoxically, MB is also known to induce beneficial effects through its oxidizing properties. For example, MB has been demonstrated to have beneficial anti-tau aggregation effects *in vitro*^8^,^9^. It does so by inducing the oxidation of cysteine residue(s) within tau. Oxidative stress response is another potential pathway towards suppression of tau accumulation and accompanying neurotoxicity^10^. However, those mechanisms were also suggested to be inconsistent with the concentration in the human brain following oral dosing^18^,^41^.

To what extent our findings in flies can be applied to humans remains to be revealed unequivocally. Based on the amount of daily food intake of a fly (1.5 ± 0.04 μL)^42^ in comparison to its body surface area (BSA), for a fly that is 2 mm in height and 0.5 mg in body weight, we estimated the human equivalent dose of MB using the following equations^43^,^44^ (Equation 1, 2, 3):
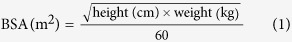

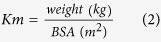

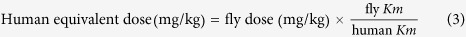


Our calculations revealed that agar containing 1 mM MB translates to a fly dose of 480 ng MB per day. This was estimated to be equivalent to around 150 mg per day for a human with a body weight of 60 kg and at a *Km* of 37. This dose is very close to the oral dosages used in clinical trials for MB^18^. Although the concentration of the active moiety of MB in the brains of *Drosophila* and humans may differ even if the intake is the same, the possible involvement of oxidation in the suppression of tau accumulation in brain is yet to be excluded.

If oxidation is involved in both the suppression of tau pathology and detrimental side effects, then safety becomes an even more important issue when developing an oxidant as a drug to relieve the burden of accumulated pathological tau. We previously observed that low levels of oxidation via alloxan suppress tau accumulation in mouse models of AD^11^. But, alloxan is diabetogenic and cannot be applied for a human use easily. MB-associated oxidation arises, in part, through photosensitization and the subsequent generation of a specific form of non-free radical ROS called singlet oxygen^23^,^24^. In the present study, we detected under light the generation of singlet oxygen from MB-treated fly homogenates (Fig. 5a) and discovered that light was required for the MB-induced reduction of tau in our fly model (Fig. 5b,c). In an ongoing search for drug candidates capable of reducing tau pathology, we identified a known compound, RB, that generates singlet oxygen and that is relatively nontoxic upon intake.

RB is a photosensitizer and food additive^34^,^35^. Like MB, in the present study RB treatment reduced tau accumulation and ameliorated climbing activity in human tau-expressing flies, but to a smaller extent (Fig. 2). Improving effects on the climbing activity by MB or RB treatment were not observed in continuous dark condition, indicating a role of photosensitive property commonly possessed by these compounds. Significant reduction of toxic effects of MB without light suggests the role of photosensitive action in the toxicity as well (Fig. 4). However, certain degree of toxicity by MB treatment without light suggests alternative way to the detriments. Unlike MB, we were unable to detect those detrimental effects as a result of RB treatment (Fig. 4). Taken together, these results suggest that RB is less potent in generating both beneficial and detrimental effects than MB at the same dose fed on flies, although it is still not excluded that the pharmacological action of two compounds is different. Nonetheless, RB but not MB ameliorated the associative memory deficits of mb;hTau fly model (Fig. 6). Therefore, we propose that singlet oxygen-generating compounds with a good safety profile may be suitable drug candidates for targeting tau therapeutically. In the application to humans, an issue is expected to arise in how to provide sufficient light to generate singlet oxygen in the brains, in which insufficient light intensity were given. We are currently exploring for a solution by combining the use of near infrared light that penetrates into the cortical surface and photosensitive compound reacting to the wavelength range of near infrared light.

## Methods

### Fly strains

All experiments were performed at 25 °C and on a 12 h day-night cycle, with 65 ± 5% room humidity unless otherwise mentioned. The flies were reared on standard cornmeal-sugar-yeast-agar medium. We obtained 2N4R human tau (hTau) cDNA in a pRK172 plasmid from Dr. Michel Goedert (Medical Research Council, UK) via Dr. Masato Hasegawa (Tokyo Metropolitan Institute of Medical Science, Japan). The hTau cDNA was subcloned into a *pUAST* vector using *Eco*RI *and Not*I restriction sites. *UAS-hTau* transgene containing a *pUAST* vector was later microinjected into *yw* fly embryos by BestGene Inc. (Chino Hills, CA, USA). Larvae that contain the *hTau* transgene were obtained from BestGene Inc. and grown to adult flies. Adult flies that carry *hTau* transgene were selected and crossed with flies that carry a third chromosome balancer TM3 (*Sb-Ser).* Adult progeny male flies and virgin female flies that carry the *hTau* transgene and TM3 balancer were crossed to establish the *yw;UAS-hTau/TM3* line. Adult *UAS-hTau* male flies were mated with virgin flies carrying the GMR*-GAL4*, *elav*^*C155*^(*elav-GAL4*), or *mb247-GAL4* driver gene in order to express hTau in eyes, pan neurons, and mushroom body neurons (mb), respectively. Newborn GMR*-GAL4/Y;hTau/+* (GMR;hTau) flies and *mb247-GAL4/Y;hTau/+* (mb;hTau) flies and 1- to 3-day-old *elav*^*C155*^*/Y;hTau/+* (ELAV;hTau) flies were used for compound treatment experiments. Wild-type fly *w*^*1118*^ was used as a control in biochemical, climbing and survival assays. Wild-type fly *yw* was mated with flies carrying the *mb247-GAL4* driver gene, and the progeny carrying the *mb247-GAL4/Y* gene (mb;yw) was used as a control in the olfactory memory assay. *elav-GAL4* was obtained from *Drosophila* Genetic Resource Center at Kyoto Institute of Technology. GMR-*GAL4* and *mb247-GAL4* were gifted from Drs. Fumiko Hirose (University of Hyogo, Japan) and Minoru Saitoe (Tokyo Metropolitan Institute of Medical Science, Japan), respectively.

### Compound treatment

MB (catalog no. 133-06962) and RB (catalog no. 184-00272) were purchased from WAKO Chemicals, Japan. These compounds were dissolved in milliQ-filtered water (Merck Millipore) to obtain 100 mM stock solution. An appropriate volume of stock solution was added to warmed (65 °C) agar and mixed in a magnetic stirrer to obtain agar containing 1 mM MB or 1 mM RB. Five milliliters of agar was poured into 19.5 cm plastic vials and allowed to cool down and solidify. The vials containing the solidified agar were covered with cotton cloth and kept at room temperature overnight in order to dry excess moisture present on the surface of solidified agar to prevent flies from sticking to it. On the following day, 25 flies expressing hTau protein were transferred to a vial containing agar with or without 1 mM compound. At least three vials for each treatment group were prepared. Each experiment was repeated more than two times, and an average value of all the experiment was calculated. Flies were kept in the same vial during experiments using GMR;hTau and mb;hTau flies. In experiments using ELAV;hTau flies, the vial was replaced with a new one in two weeks because climbing deficits induced by hTau expression in neurons made those flies more susceptible to get stuck in their feces. Total MB or RB treatment time was one month or two weeks unless otherwise stated. The vials containing flies were either kept under normal (12 h:12 h) light cycle (“light” condition) or under continuous darkness (“dark” condition).

### Biochemistry

Using sharp tweezers, we dissected the heads of 20 flies and collected them in a 1.5 ml ultracentrifuge tube (Beckman Coulter, Brea, CA, USA) containing ice-cold PBS, which was removed before the tubes were placed in a −80 °C deep freezer. TBS-soluble protein and sarkosyl-insoluble fraction was extracted following the procedures developed by Drs. Greenberg and Davies^25^ with minor changes. TBS-soluble protein was extracted from the fly heads by adding Tris-buffered saline (TBS; 10 mM Tris, 150 mM NaCl [pH 7.4], 1 mM EDTA, 1 mM EGTA) to each tube containing 20 heads (5 μl/head) and by homogenizing in a microhomogenizer (Physcotron, Micro Homogenizer, Microtec Co., LTD., Japan) for 45 s. The homogenate was centrifuged at 23,000 rpm (23,699 *g*) at 2 °C for 25 min in an ultracentrifuge (Optima MAX-TL Ultracentrifuge, Beckman Coulter, Brea, CA, USA). After centrifugation the supernatant was collected as the TBS-soluble fraction, and the pellet was resuspended in the same volume (5 μl/head) of 10% sucrose buffer (10 mM Tris, 800 mM NaCl [pH 7.4], 1 mM EGTA, 10% Sucrose). The resuspended solution was homogenized for 45 s and centrifuged again at 23,000 rpm (23,699 *g*) at 2 °C for 25 min. The resulting supernatant was mixed with 1/10 volume of 10% sarkosyl solution in polycarbonate ultracentrifuge tubes and incubated for 3 h at room temperature. The tubes were then centrifuged at 100,000 rpm (417,200 *g*) at 4 °C for 1 h. The supernatant was collected as the sarkosyl-soluble fraction, and the pellet was resuspended in 2x Laemli buffer (120 mM Tris [pH 6.8], 10% 2-mercaptoethanol, 4% SDS, 20% glycerol, and 0.02% bromophenol blue) by vortexing. The resulting suspension was collected and frozen as the sarkosyl-insoluble fraction. For the extraction of RIPA-soluble fraction, the pellet after collecting TBS-soluble fraction was resuspended in the volume (2.5 μl/head) of mRIPA buffer (50 mM Tris [pH7.4], 1% NP-40, 0.25% Sodium deoxycholate, 150 mM NaCl, 1 mM EGTA). The resuspended solution was sonicated by a handy sonicator (Smurt NR-50 M, Ultrasonic Homogenizer, Microtec Co., LTD., Japan) for 15 s on ice and centrifuged again at 23,000 rpm (23,699 *g*) at 2 °C for 25 min. The resulting supernatant was collected as the RIPA-soluble fraction. All lysis buffers contained protease inhibitors (5 μg/mL pepstatin, 5 μg/mL leupeptin, 2 μg/mL aprotinin, and 0.5 mM 4-(2-aminoethyl) benzenesulfonyl fluoride hydrochloride); phosphatase inhibitors (1 μM okadaic acid, 1 mM Na_3_VO_4_, and 1 mM NaF); and deacetylase inhibitors (1 μM trichostatin A and 5 mM nicotinamide).

Protein levels in the TBS-soluble, RIPA-soluble, and sarkosyl-insoluble fractions were analyzed by western blotting. Samples were subjected to SDS-PAGE and immunoblotted with pan-human tau antibodies (JM, 1:1000, a kind gift from Dr. Akihiko Takashima [National Center for Geriatrics and Gerontology, Japan]; and Tau5, 1:5000, catalog no. AHB0042, Biosource, San Diego, CA, USA). JM is a rabbit polyclonal antibody raised against recombinant human tau that recognizes both phosphorylated and non-phosphorylated tau^45^. Immunoreactivity of hTau with these pan-tau antibodies can be distinguished from that of endogenous fly tau by the molecular size, as 2N4R hTau (65 kDa) is larger than fly tau (50 kDa). We have generated a monoclonal antibody specific to Drosophila tau, Dtau. Anti-β-actin (Cell Signaling Technology, Boston, MA), Nrf2 (Santa Cruz Biotechnology, Dallas, TX), and APG5L/ATG5 (Abcam, Cambridge, MA) antibodies were purchased from respective companies. Imaging and visual analysis of the immunoreactive bands were performed with a computer-linked LAS-4000 Bio-Imaging Analyzer System (Fujifilm, Tokyo, Japan). Immunoreactivity was quantified by using ImageJ 1.46r software^46^.

### Climbing assay

Flies instinctively climb upward on a vertical surface against gravity, a phenomenon termed negative geotaxis. We conducted a climbing assay to investigate whether ELAV;hTau flies’ native climbing ability is affected by hTau expression in neurons, and also to determine whether MB or RB treatment (see **Compound treatment** in **Methods**) affects climbing deficits in these flies and whether it affects wild-type flies.

Twenty-five flies were transferred into an empty vial. The vial was tapped on a rubber pad gently to bring all the flies down to the bottom, or floor, of the vial. Normally, flies start climbing up onto the inner wall of the vial and continue until they reach the top. This behavior was video recorded and quantified. We calculated the percentage of the number of flies that climbed 3 cm or higher above the bottom of the vial within 10 s. Experiments were performed three times for each batch of flies.

For experiments investigating the relationship between tau accumulation and climbing ability, we separated out flies according to climbing ability after one month of either 1 mM MB, 1 mM RB, or control agar (see **Compound treatment** in **Methods**). Specifically after treatment, we collected ELAV;hTau flies that could climb and flies that could not climb, separating them by a partition in the vial located 3 cm above the bottom of vial. A narrow opening located 3 cm above the bottom of an empty vial was made by cutting it transversely until half of it was open. To separate and collect the two groups of flies, we inserted a thick, U-shaped paper card into the narrow opening of the vial, so that the flies that could climb 3 cm were confined to the top part of the vial and flies that could not climb were confined to the bottom part of the vial. Material for analysis of protein fractions was collected and prepared as described above. The climbing assay was also performed to investigate any possible adverse effects of compound treatment on the climbing ability of wild-type *w*^*1118*^flies.

### Survival assay

A survival assay was performed using male wild-type *w*^*1118*^flies. All vials were kept in a horizontal position, and the agar was changed every two weeks. The number of dead flies was counted three times a week. Flies that escaped and flies that stuck onto the surface of the agar and died did not contribute to the survival count data. After all the flies died, a survival curve was plotted. By using Prism 6 software (GraphPad Software), the log-rank Mantel-Cox test was performed to determine significant differences among groups.

### Singlet Oxygen measurement

Amount of singlet oxygen generated in fly homogenate was measured. GMR;hTau flies were treated with either 1 mM MB agar or control agar for two weeks. 100 μL of 6 M guanidine-HCl containing reagent buffer (100 mM Tris-HCl [pH 7.8], 4 mM Na EDTA) was added to a tube with five anesthetized flies on ice. After homogenizing in a microhomogenizer (Physcotron, Micro Homogenizer, Microtec Co., LTD., Japan) for 45 s, the sample tubes were centrifuged at 10,000 g at 2 °C for 5 min. 48 μL of each sample was mixed with 50 μL of the reagent buffer and 2 μL of either methanol (Wako, Japan) or 5 mM Singlet Oxygen Sensor Green (SOSG) agent (Molecular Probes, USA) dissolved in methanol. 80 μL of each sample mixture was poured into a well of 96-well plate (Black Microtiter Plate, Thermo Scientific, Finland). The fluorescence signal was measured at excitation 488 nm and emission 525 nm by a spectrophotometer (SpectraMax M5^e^, Molecular Devices, USA). The plate covered with transparent lid was placed under white LED light during incubation.

### Olfactory memory assay

To further clarify the effect of RB treatment on olfactory memory deficits previously reported in mb;hTau flies^22^, we tested short-term memory of flies treated with or without 1 mM RB for one week following the procedures developed by Dr. Tully^47^, with minor changes. Two aversive odors for flies, 3-octanol (OCT) and 4-methylcylohexanol (MCH), were sequentially delivered, counterbalanced, to approximately 100 flies for 1 min each, separated by a 45 s interval. When the flies were exposed to the first odor (OCT or MCH), they were also subjected to 1.5 s pulses of 60 V DC electric shocks every 5 s. Then to assess memory for the odor, we placed flies at the choice point in a T-maze, where each odor was delivered to either one of the chambers. After two minutes, we sorted flies according to how they responded. Flies that moved into the chamber with the electric shock-associated odor were separated from the flies that moved into the other chamber. Olfactory memory was estimated by calculating a performance index (PI). This was defined so that a 50:50 distribution between the two odors (no memory) yielded a PI of zero and a 0:100 distribution away from the odor associated with electric shock yielded a performance index of 100.

Prior to olfactory memory testing, we used the mb;hTau flies used in the memory test to determine to what extent flies avoided aversive odors or electric shocks. Approximately 100 flies were placed at the choice point of T-maze and allowed to choose between an aversive odor (OCT or MCH) and fresh air (odor avoidance) or between electric shock and no electric-shock (shock avoidance) conditions. PI for odor avoidance and shock avoidance were determined the same way as in the olfactory memory test. We confirmed that the PI of OCT avoidance and MCH avoidance were not significantly different.

### Statistics

We used PRISM6 (GraphPad Software, La Jolla, CA, USA) and two-tailed Mann-Whitney tests to assess control and treatment group differences unless otherwise stated. Statistical significance was set at *P* < 0.05.

## Additional Information

**How to cite this article**: Sheik Mohideen, S. *et al.* Nontoxic singlet oxygen generator as a therapeutic candidate for treating tauopathies. *Sci. Rep.* **5**, 10821; doi: 10.1038/srep10821 (2015).

## Supplementary Material

Supplementary Information
