# Supplementary material for: Nontoxic singlet oxygen generator as a therapeutic candidate for treating tauopathies
Source: Sci Rep. 2015 Jun 1;5:10821. doi: 10.1038/srep10821 (PMC4450544; doi:10.1038/srep10821)
Supplement: Supplementary Information [file srep10821-s1.pdf]

Nontoxic singlet oxygen generator as a therapeutic candidate for treating tauopathies

Sahabudeen Sheik Mohideen, Yasutoyo Yamasaki, Yasuhiro Omata, Leo Tsuda, Yuji Yoshiike

Supplementary Figures

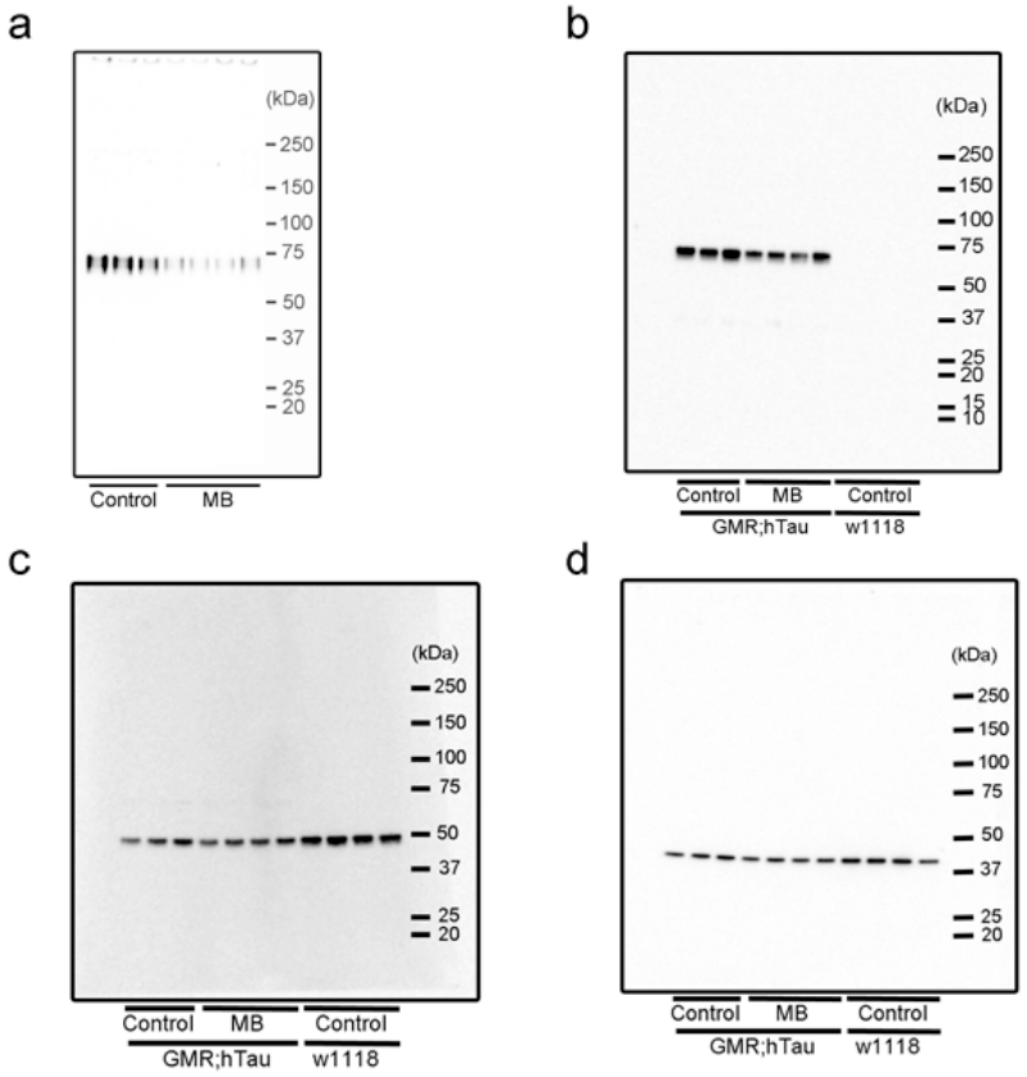

Supplementary Figure S1. MB treatment reduces tau levels in sarkosyl-insoluble and TBS-soluble fractions from GMR;hTau flies. After one-month MB (1 mM) treatment, both fractions were prepared from the heads and analyzed by Western blot. (a) Full-length blot of sarkosyl-insoluble fraction probed with JM antibody. (b) Full-length blot of TBS-soluble fraction probed with tau5 antibody. (c) Full-length blot of TBS-soluble fraction probed with Dtau antibody. (d) Full-length blot of TBS-soluble fraction probed with β-actin antibody.

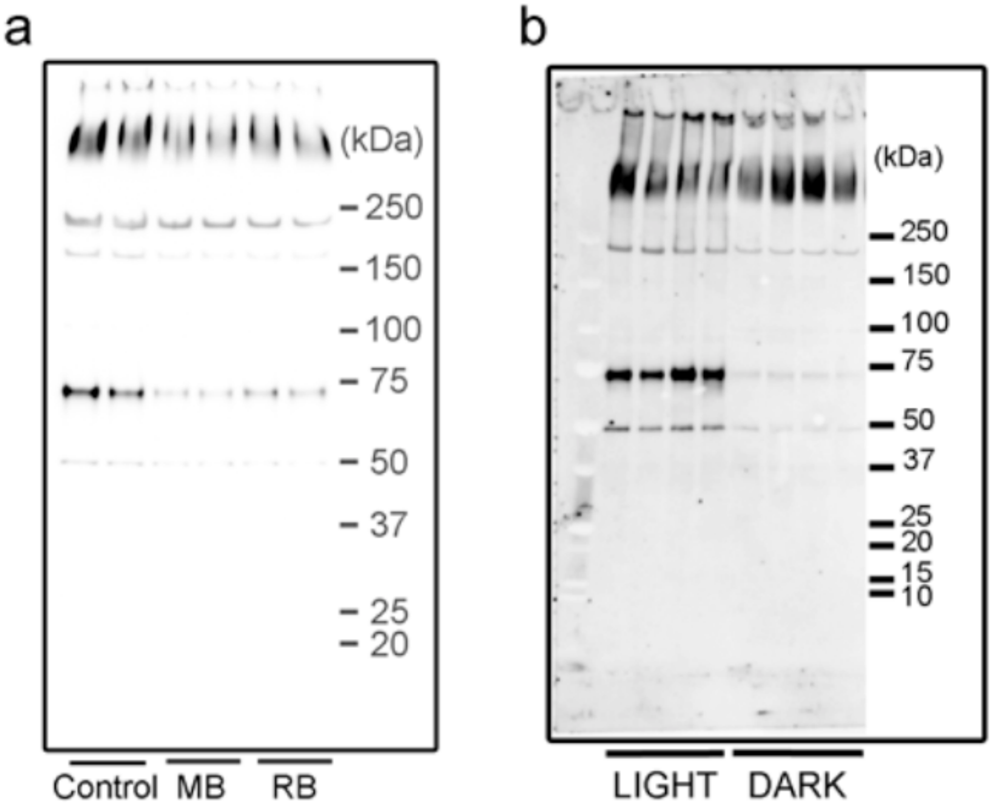

Supplementary Figure S2. Compound treatments and light affects tau accumulation in sarkosyl-insoluble fractions from ELAV;hTau flies. (a) Full-length western blot showing JM immunoreactivity of sarkosyl-insoluble fractions derived from the heads of control (untreated), MB-treated, and RB-treated ELAV;hTau flies. (b) Full-length western blot showing JM immunoreactivity of sarkosyl-insoluble fractions derived from the heads of ELAV;hTau flies that were kept in normal "light" (12h:12h) cycle or continuous "dark" condition.

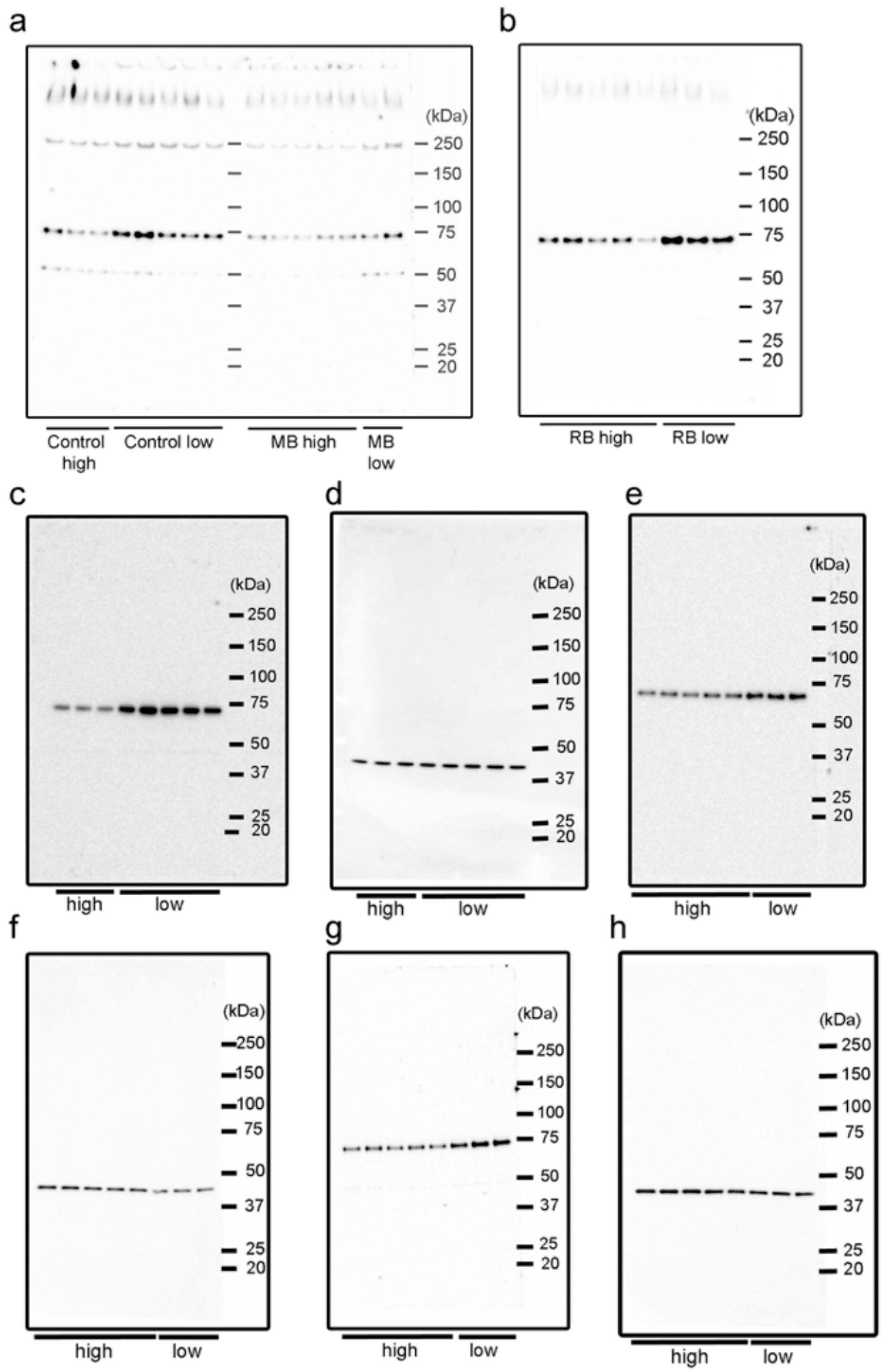

Supplementary Figure S3. Climbing deficits in ELAV;hTau flies were correlated with the accumulation of insoluble and soluble tau. Flies that climbed higher than 3 cm from the bottom of the vial (high) and flies that could not climb (low) were

separated and counted before their heads were dissected for biochemical analysis. (a) Full-length western blot of sarkosyl-insoluble fractions from “high” and “low” climbing flies in control untreated and MB-treated groups probed with JM. (b) Full-length western blot of sarkosyl-insoluble fractions from “high” and “low” climbing flies in RB-treated groups probed with JM. (c) Full-length western blot of TBS-soluble fractions from “high” and “low” climbing flies in control untreated group probed with tau5. (d) Full-length western blot of TBS-soluble fractions from “high” and “low” climbing flies in control untreated group probed with β-actin. (e) Full-length western blot of TBS-soluble fractions from “high” and “low” climbing flies in MB-treated group probed with tau5. (f) Full-length western blot of TBS-soluble fractions from “high” and “low” climbing flies in MB-treated group probed with β-actin. (g) Full-length western blot of TBS-soluble fractions from “high” and “low” climbing flies in RB-treated group probed with tau5. (h) Full-length western blot of TBS-soluble fractions from “high” and “low” climbing flies in RB-treated group probed with β-actin.

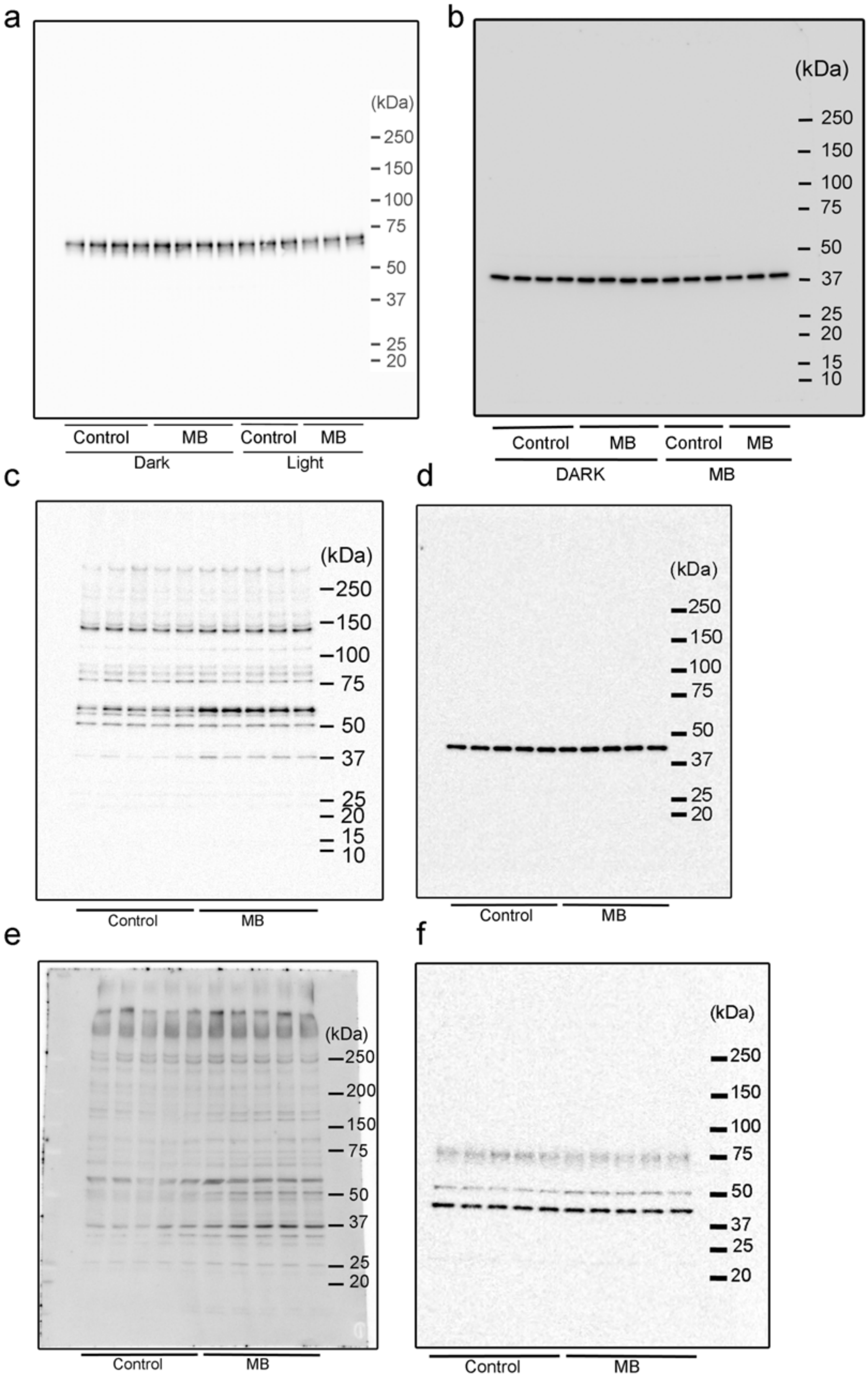
a
(kDa)
250
150
100
75
50
37
25
20
Control
MB
Control
MB
Dark
Light
b
(kDa)
250
150
100
75
50
37
25
20
15
10
Control
MB
Control
MB
DARK
MB
c
(kDa)
250
150
100
75
50
37
25
20
15
10
Control
MB
d
(kDa)
250
150
100
75
50
37
25
20
Control
MB
e
(kDa)
250
200
150
75
50
37
25
20
Control
MB
f
(kDa)
250
150
100
75
50
37
25
20
Control
MB

Supplementary Figure S4. MB fails to reduce tau in GMR;hTau flies housed under continuously dark condition while MB increases Nrf2 and ATG5 in GMR;hTau flies under normal light cycle condition. GMR;hTau flies were treated with 1 mM MB agar or control agar under either continuous darkness or normal light cycle conditions (12h:12h). After two weeks of treatment, fly heads were dissected and TBS-soluble or RIPA-soluble fractions were analyzed by western blot. (a) Full-length western blot of TBS-soluble fractions probed with tau5. (b) Full-length western blot of TBS-soluble fractions probed with β-actin. (c) Full-length western blot of RIPA-soluble fractions probed with Nrf2. (d) Full-length western blot of RIPA-soluble fractions probed with β-actin. (e) Full-length western blot of TBS-soluble fractions probed with ATG5. (f) Full-length western blot of TBS-soluble fractions probed with β-actin. Samples from (c) to (f) were originated from flies kept under normal light cycle condition.
